# Preptin: A New Bone Metabolic Parameter?

**DOI:** 10.3390/metabo13090991

**Published:** 2023-09-04

**Authors:** Maria-Christina Ungureanu, Stefana Catalina Bilha, Mihai Hogas, Cristian Velicescu, Letitia Leustean, Laura Claudia Teodoriu, Cristina Preda

**Affiliations:** 1 Endocrinology Department, “Grigore T. Popa” University of Medicine and Pharmacy, 700115 Iasi, Romania; maria.ungureanu@umfiasi.ro (M.-C.U.);; 2 Physiology Department, “Grigore T. Popa” University of Medicine and Pharmacy, 700115 Iasi, Romania; 3 Surgery Department, “Grigore T. Popa” University of Medicine and Pharmacy, 700115 Iasi, Romania

**Keywords:** preptin, insulin resistance, osteoblasts, bone, bone mineral density

## Abstract

Preptin is a 34-aminoacid peptide derived from the E-peptide of pro-insulin-like growth factor 2 (pro-IGF2) that is co-secreted with insulin and upregulates glucose-mediated insulin secretion. High serum preptin levels were described in conditions associated with insulin resistance, such as polycystic ovary syndrome and type 2 diabetes mellitus (T2M). Insulin and also IGF2 are known to be anabolic bone hormones. The “sweet bone” in T2M usually associates increased density, but altered microarchitecture. Therefore, preptin was proposed to be one of the energy regulatory hormones that positively impacts bone health. Experimental data demonstrate a beneficial impact of preptin upon the osteoblasts. Preptin also appears to regulate osteocalcin secretion, which in turn regulates insulin sensitivity. Preptin is greatly influenced by the glucose tolerance status and the level of physical exercise, both influencing the bone mass. Clinical studies describe low serum preptin concentrations in osteoporosis in both men and women, therefore opening the way towards considering preptin a potential bone anabolic therapy. The current review addresses the relationship between preptin and bone mass and metabolism in the experimental and clinical setting, also considering the effects of preptin on carbohydrate metabolism and the pancreatic–bone loop.

## 1. Introduction

Preptin is the youngest member of the insulin family [1]. A 34-aminoacid peptide derived from the E-peptide of pro-insulin-like growth factor 2 (pro-IGF 2), preptin was first isolated in 2001 by Buchanan et al. [2] from rat pancreatic β-cell islet granules. Preptin is co-secreted with insulin and enhances glucose-mediated insulin secretion [2].

Further on, in vitro studies described a dose-dependent increase in insulin secretion caused by preptin in high glucose conditions, but not in normal conditions. The effect was reported to be similar to that of glibenclamide [3]. Cheng et al. [3] reported autocrine actions of preptin by locally activating the IGF2 receptor (IGF2R) via the phospholipase C/protein kinase C pathway, thus provoking calcium-dependent insulin secretion [3].

However, the systemic effects of preptin on glucose metabolism remain largely undetermined. Multiple sources of preptin secretion besides beta cells, such as the kidneys, liver, salivary gland and mammary tissue, have been found since its discovery [1]. Female preptin knockout mice exhibit reduced glucose-enhanced insulin secretion, but not males [4]. Early clinical studies reported higher plasma preptin concentrations in patients with type 2 diabetes mellitus (T2M) compared to patients with impaired glucose tolerance and controls, respectively. Lower preptin levels were found in men compared to women [5]. Preptin appears to regulate metabolic homeostasis via glucose-mediated insulin secretion enhancement and is, therefore, linked to insulin resistance [1]. Thus, preptin was found to be elevated in conditions associated with insulin resistance, such as polycystic ovary syndrome (PCOS), gestational diabetes mellitus and T2M [5,6,7].

Despite being associated with insulin resistance, an increased fat mass has beneficial bone effects (Reid, 2010) [8]. While the increased mechanical loading favors bone formation, adipokines and also pancreatic and gut hormones were also proposed to mediate the relationship between fat and bone. The importance of nutritional hormones in maintaining bone mass was, thus, recognized. Insulin has anabolic bone effects, and hyperinsulinemia contributes to increased bone mineral density (BMD) [9]. Similar to insulin, insulin-like growth factor 1 (IGF1) and IGF2, preptin was recently proposed to have anabolic bone activity [9,10,11]. The current manuscript aims to review experimental and clinical data investigating the bone actions of preptin. As preptin is increased in obesity and states of hyperinsulinemia, its metabolic effects that could interfere with bone metabolism are also discussed.

## 2. Methods

We searched the PubMed electronic database from inception up to July 2023 using the following keywords: “preptin”, “bone”, “bone mineral density”/”BMD”, “bone turnover” and “calcium”. Original articles reporting data regarding the relationship between preptin and bone density and/or metabolism or calcium and phosphate metabolism were included.

Additionally, studies reporting the relationship between preptin and glucose metabolism are also briefly discussed due to the potential implication of preptin in the relationship between nutrition, energy metabolism and bone mass. Relevant references from the selected articles were also searched manually.

## 3. Preptin, Insulin Resistance and Bone Metabolism

### 3.1. Insulin Resistance: The Contradictory Status of Preptin in PCOS

T2DM patients and individuals exhibiting impaired glucose tolerance assessed via the 2 h glucose tolerance test (OGTT) have significantly higher serum preptin concentrations compared to individuals with normal glucose tolerance [5,12]. Preptin is also associated with vascular complications of T2M, such as diabetic nephropathy and retinopathy, respectively [13,14].

As preptin levels were independently associated with metabolic parameters, such as diastolic blood pressure, triglycerides, high-density lipoprotein (HDL) cholesterol and free fatty acids in the study of Yang et al. [5], and with Homeostatic Model Assessment for Insulin Resistance (HOMA-IR), but not fasting insulin [12], preptin could be linked to insulin resistance and it was, therefore, further assessed in women with PCOS. However, the data are still contradictory regarding preptin status in PCOS, a common endocrine disorder characterized by hyperinsulinemia, insulin resistance, hyperandrogenism and often weight gain [15].

Celik et al. [6] in 2011 were the first to investigate serum preptin levels in PCOS women and reported significantly higher concentrations in women suffering from PCOS compared to controls (823.2 ± 140.7 pg/mL versus 324.9 ± 147.3 pg/mL, *p* < 0.001). Preptin correlated with both fasting plasma insulin and HOMA-IR and Ferriman–Gallwey hirsutism score, but not with body mass index (BMI) [6].

One year later, in the study of Bu et al. [12] serum preptin concentrations had the tendency to be higher in women with PCOS compared with patients without PCOS, but the difference did not reach statistical significance; nonetheless, preptin levels differed only according to glucose tolerance status. In multiple regression analysis, preptin was an independent predictor of glucose tolerance, but not of PCOS status, after adjusting for anthropometric variables, age, blood pressure, lipid profile and sex hormone concentrations. However, none of the patients included in the analysis had T2M [12].

The variation of serum preptin levels according to BMI (<25 kg/m^2^ versus ≥25 kg/m^2^) in PCOS compared to healthy women was further assessed [16]. Overweight PCOS women had higher preptin levels compared to overweight controls, but the statistical significance was not reached, probably due to the very small sample size (*n* = 20 subjects in each group) [16]. When assessed in larger cohorts (73 PCOS women and 61 controls matched for nutritional status), serum preptin was significantly higher in PCOS women compared to controls [17]. However, the study failed to find any significant correlations between preptin and metabolic and hormonal parameters, respectively. Nevertheless, in pooled data regression analysis, preptin levels ≥7.5 ng/mL and free androgen index ≥5 independently discriminated between the two groups [17]. However, preptin values [17] were higher than previously reported in PCOS patients [6,12], potentially contributing to the less clear lack of association between preptin and biochemical parameters.

A recent meta-analysis [18] in 2023 including eight studies of moderate quality (Newcastle–Ottawa Scale score between 5 and 7) that assessed preptin levels in PCOS concluded that serum preptin is significantly higher in PCOS patients compared to controls, with significant heterogeneity between studies possibly due to age, BMI and degree of insulin resistance. Preptin concentrations were higher in women with BMI < 25 kg/m^2^ and in women with insulin resistance displaying HOMA-IR > 3. Thus, the association between preptin and PCOS appears to be influenced by insulin resistance [18]. In the same direction, Ozkan et al. [19] previously reported significant variations of preptin according to the BMI category in the general adult population: serum preptin concentrations were higher in both low-weight and overweight/obese individuals compared to normal-weight subjects, respectively [19].

While the main feature associated with preptin increase seems to be the presence of insulin resistance, physical exercise improves both insulin sensitivity and downregulates preptin concentrations, as demonstrated in male patients with prediabetes [20]. Both high-intensity interval training and continuous endurance training for 12 weeks significantly decreased preptin levels together with HOMA-IR [20]. Therefore, when assessing the relationship between preptin and bone mass, the level of physical exercise should be considered.

### 3.2. The Dual Role of Insulin Resistance in Bone Mass and Metabolism

Insulin and bone are interconnected. Insulin stimulates human osteoblast differentiation [21], while impaired insulin receptor signaling in osteoblasts hinders bone mass acquisition [22]. Insulin also stimulates osteocalcin production in the osteoblast, which in turn closes the loop by regulating insulin secretion and insulin sensitivity [22,23]. In vitro, insulin also promotes collagen and ALP synthesis, glucose uptake and inhibits osteoclast activity [24]. On the other hand, prolonged hyperglycemia is detrimental for bone: it favors adipogenic instead of osteogenic differentiation [25], while advanced glycation end products promote oxidative stress, inflammation and low bone turnover [26,27].

The bone deleterious effects of insulinopenia are very-well seen in type 1 diabetes mellitus (T1D), where low bone mass accrual is seen after disease onset [28]. T2M patients are reported to have increased BMD [29], probably due to increased BMI, which is a protective factor against osteoporosis in all populations due to the mechanical loading [30]. Our group also found BMI to be a positive independent predictor in T2M [31]. Hyperinsulinism secondary to insulin resistance may contribute to this finding. However, cortical bone microarchitecture is altered in T2M patients [32], which exhibit reduced trabecular bone score despite increased BMD [29]. In advanced disease, microvascular damage, insulinopenia and visceral adiposity negatively impact bone mass and turnover [26]. The “sweet bone” appears to be insulin resistant. Mice fed a high fat diet have bone insulin resistance, and hence a low bone turnover and low osteocalcin [33]. A low osteocalcin favors glucose intolerance, according to the pancreatic–bone loop [33].

In the clinical setting, increased insulin levels and insulin resistance have been associated with higher areal and volumetric BMD in postmenopausal women, elderly adults and PCOS women [34,35,36,37]. Insulin resistance was, however, inversely related to the trabecular area at a distal radius, the cortical area at the radial and tibial shafts and also with cortical thickness at the tibia [38]. Therefore, despite an increased in BMD, insulin resistance is associated with reduced bone cortical thickness, bone strength and bone turnover (low osteocalcin production as a consequence of osteoblast insulin resistance) [24,39].

Preptin rises in hyperinsulinism and insulin resistance states and was also reported to be anabolic on bone metabolism. What is known up to the writing of this manuscript about the bone effects of preptin is discussed below. How preptin integrates in the pancreatic–bone loop still remains to be elucidated.

## 4. The Impact of Preptin on Bone and Calcium Metabolism

### 4.1. What Do We Know from Experimental Data

Preptin stimulates osteoblastic proliferation and increases mineralization in a concentration-independent manner in osteoblast precursor cells [40]. The Wnt/β-catenin pathway plays an essential role in osteoblast differentiation and survival, and thus in bone mass maintenance. Activation of Wnt/β-catenin canonical pathway upregulates the expression of the bone-related transcription factor RUNX2, which is required for the onset of osteoblast differentiation [41,42]. β-catenin also maintains the viability of the osteocytes, which are necessary for bone remodeling [43,44]. Moreso, the Wnt/β-catenin pathway is responsible for mechanical loading stimuli transduction to bone cells [45]. Dickkopf 1 (DKK1) is a Wnt receptor antagonist that hampers the interaction between Wnt proteins and their receptors [46]. Preptin upregulated β-catenin and RUNX2 expression in vitro [40]. Preptin treatment of osteoblast precursor cell line also increased osteocalcin expression, a downstream target of RUNX2 signaling [40]. The osteogenic effect of preptin in vitro is attenuated osteoblast precursor cells treated with DKK1; thus, preptin seems to promote the proliferation and differentiation of osteoblasts via the Wnt/β-catenin pathway [40].

**Figure 1:**
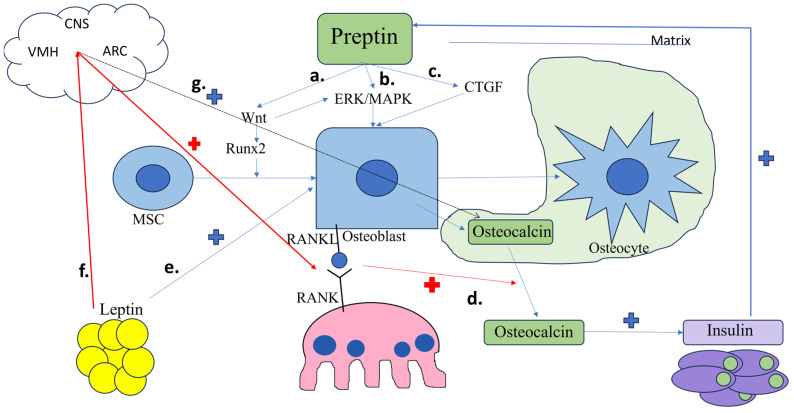
Proposed mechanisms for the anabolic action of preptin: a. activation of Wnt/β-catenin and RUNX2, which promotes osteoblast differentiation and proliferation; b. activation of ERK/MAPK signaling, which is also activated by Wnt, leading to osteoblast differentiation and activation; c. upregulation of CTGF, which also activates ERK, promoting bone formation and mineralization; d. coupling of RANKL from osteoblasts to RANK on osteoclasts activates resorption and osteocalcin release from the bone matrix, which in turn stimulates insulin secretion, which exerts anabolic bone effects directly and via preptin; e. leptin secretion from the adipocytes directly stimulates osteoblast differentiation and proliferation; f. leptin indirectly stimulates bone resorption by activating β2 adrenergic receptors via the CNS; g. leptin indirectly offsets osteocalcin decarboxylation and release via increasing *Esp* gene expression. Blue cross = anabolic pathways, red cross = resorption pathways. RC = the arcuate nucleus, CNS = central nervous system, CTGF = connective tissue growth factor, MSC = mesenchymal stem cell, RANK = receptor activator of nuclear factor κB, RANKL = RANK-ligand, VMH = the ventromedial nucleus of the hypothalamus.

Cornish et al. [47] demonstrated that preptin stimulates proliferation of rat osteoblast-like cells and also bone nodule formation via bone matrix deposition and mineralization in a dose-dependent manner. However, preptin did not affect osteoclast development and activity [47]. The mitogenic preptin-induced signaling in osteoblasts is mediated by the p42/p44 MAP kinases (MAPK) [47], also known as Erk 2 and Erk1 [48]. ERK/MAPK are activated by extracellular mitogen stimulation, leading to intracellular signal transduction to the nucleus and thus promoting the early differentiation of osteoblast precursors [48,49,50]. ERKs are also activated by the Wnt signaling pathway [51], also stimulated by preptin [40] (Figure 1). More so, preptin was also reported to have antiapoptotic effects upon osteoblasts in vitro. In vivo, peptin administration to adult mice significantly increased mineralization and bone surface compared to controls in a dose-dependent manner [47].

The proliferative effect of preptin was also confirmed in human osteoblasts, where it also increases alkaline phosphatase (ALP) in a dose-dependent manner. The response of human osteoblasts to preptin was reported to be bell-shaped [52]. Preptin treatment also leads to a dose-dependent increase in connective tissue growth factor (CTGF) [52]. CTGF promotes osteogenesis during active growth, bone modelling or fracture healing [53]. CTGF is a matricellular protein contained in the extracellular matrix [54]. Hendesi et al. [54] demonstrated that osteoblasts attach to CTGF, forming focal adhesions that activate the ERK signaling pathway. Bone nodule formation and matrix mineralization are then enhanced, and osteogenic differentiation is promoted. Osteocalcin binding to RUNX2 is also increased [54]. Blocking either the MAPK signal pathway or CTGF expression hampers the proliferative response to osteoblasts induced by preptin [52]. Therefore, the anabolic bone effect of preptin is, at least partially, mediated via CTGF induction [53] and ERK activation (Figure 1).

A very recent article published in 2023 reported decreased serum preptin levels, as well as increased bone turnover markers, in ovariectomized rats compared to controls [55]. However, serum preptin significantly increased in ovariectomized rats (1) treated with estradiol, (2) that practiced moderate exercise training or (3) that were treated with the anti-osteoporotic drug alendronate. The highest preptin concentrations were found in ovariectomized rats receiving estradiol and also performing exercise training, although the values were still lower compared to sham. Similarly, cortical bone thickness, trabecular bone thickness and osteopontin were markedly decreased after ovariectomy, but increased in ovariectomized rodents treated either with estradiol, or with alendronate [55]. BMI was higher in ovariectomized rats and significantly decreased in a similar manner when anti-osteoporotic therapies were applied, either estradiol or exercise training or both [55]. Preptin appears to mirror both metabolic and bone turnover and architecture changes associated with menopause in animal models. Whether a direct connection is involved remains to be established. The authors launched the hypothesis that estradiol may regulate the dynamics of peptin secretion [55]. Indeed, Root-Bernstein et al. [56] described estradiol to interfere with insulin binding to its receptor, thus producing insulin resistance, which is known to be associated with a mount in preptin [57,58]. Increased preptin levels after exercise training have been reported by other authors as well [59]. Moreover, preptin levels were also reported to decrease in both hypothyroidism and hyperthyroidism [60], two conditions associated with alterations in glucose homeostasis [61].

When compared to insulin, IGF1 and IGF2, preptin stimulated human osteoblasts cell proliferation at a lower level compared to IGFs, but to a greater extent than insulin, for which a proliferative effect was not observed on the short-term [62]. While higher ALP activity was encountered in human osteoblasts treated with IGFs, but not with preptin, the highest mineralization area was found in osteoblasts treated with peptin compared to IGFs or insulin [62]. Regarding the effect upon bone resorption activity, IGF1 was far more potent compared to IGF2, insulin and preptin in the study performed by Bosetti et al. [62]. IGF1 had the highest induction effect on osteoclast activity; also, when balancing the anabolic versus catabolic effect of IGF1 upon bone, the catabolic effect was more prevalent. Nevertheless, IGF1 also had the greatest activity upon osteoblast proliferation and differentiation, when assessed together with IGF2, preptin and insulin. IGF1 has the highest bone remodeling activity among them, while preptin also promotes bone formation to a lesser degree. Insulin did not regulate bone formation in the short term [62]. However, insulin was reported to be anabolic in the long-term [63].

The results from the experimental studies are summarized in Table 1.

### 4.2. Preptin and Bone Mass and Metabolism in The Clinical Setting

Li et al. [64] were among the first to evaluate the relationship between preptin and BMD in the clinical setting. In a cohort of 133 normal-weight elderly men, preptin positively correlated with lumbar spine, neck and total hip BMD, respectively, independently of age and BMI. Serum preptin was significantly lower in patients with osteoporosis, compared to the osteopenia and control group (5.1 ± 0.69 versus 7.09 ± 1.36 versus 10.11 ± 1.61, *p* < 0.05 for all) [64]. Preptin also significantly correlated with bone formation markers bone ALP, procollagen type 1 amino-terminal propeptide (P1NP) and osteocalcin, with the strongest association being found between preptin and osteocalcin (r = 0.699, *p* < 0.001) after adjusting for age and BMI. However, no relationship was found between preptin and osteoclast parameter tartrate-resistant acid phosphatase 5b (TRAP 5b) [64]. Bone formation markers were also reduced in elderly men with osteoporosis compared to controls [64], suggesting reduced bone turnover consistent with senile osteoporosis [65]. Elderly men had low BMD, low bone turnover and also low serum preptin concentrations [64]. The study opened the way towards considering preptin a target for increasing bone formation. Clinical studies investigating the relationship between preptin and BMD are depicted in Table 2.

El-Eshmawy et al. [57] reported an opposite, negative, association between preptin and osteocalcin in overweight and obese subjects, which were reported to have higher preptin concentrations, but lower levels of osteocalcin compared to controls. Fasting insulin and HOMA-IR were positive independent predictors of preptin and also negative independent predictors of osteocalcin together with the BMI (Table 2) [57].

Indeed, higher osteocalcin levels were reported to be associated with greater insulin sensitivity, lower BMI and lower systolic blood pressure [66,67], while T2M and obesity were associated with lower osteocalcin concentrations [66,68]. The active, undercarboxilated form of osteocalcin synthesized by osteoblasts exhibits various endocrine functions, among which are the regulation of insulin synthesis and secretion and also the increase in insulin sensitivity [69].

In the absence of a direct feedback tuning between insulin and osteocalcin, the latter appears to be regulated by leptin secreted from the adipocytes. Bone resorption leads to undercarboxylation of osteocalcin, its release from the mineralized bone matrix, thus allowing osteocalcin to enhance insulin production and sensitivity. On the other hand, insulin signaling in the osteoblasts upregulates osteocalcin release via osteoblast-dependent activation of osteoclasts. In contrast, leptin secreted by the adipocytes stimulates the sympathetic tone via the central nervous system (CNS), indirectly promoting bone resorption but also favoring *Esp* expression on osteoblasts from animal models—offsetting the activation and release of osteocalcin from the bone matrix (Figure 1) [70,71,72]. This may explain the dual relationship between osteocalcin and preptin reported in the literature: while preptin co-secreted with insulin stimulates osteocalcin production, the hyperleptinemia seen in obesity may counteract this mechanism and downregulate serum osteocalcin [8,73]. However, both hyperinsulinemia and hyperleptinemia are reported to be anabolic for the bone, with leptin also having direct osteoblast differentiating and proliferation actions [8,74,75]. Where preptin fits in all this complex interaction between energy metabolism and bone still remains to be clarified.

**Table 2:** Clinical studies investigating the relationship between preptin, BMD and bone and calcium metabolism.

Research	Study Group	BMI (kg/m^2^)	Preptin Concentration	Outcome
Preptin and BMD				
Li et al. [64] 2013	52 elderly men with osteoporosis	22.34 ± 1.84 *	5.1 ± 0.69 ng/mL *	Preptin (whole-group): LS (r = 0.595, *p* < 0.001), FN (r = 0.422, *p* < 0.001) and total hip BMD (r = 0.335, *p* < 0.001), after adjusting for age and BMI B-ALP (r = 0.212, *p* = 0.014), osteocalcin (r = 0.699, *p* < 0.001), P1NP (r = 0.266, *p* = 0.002)
	52 elderly men with osteopenia	22.79 ± 1.29 *	7.09 ± 1.36 ng/mL *	
	31 aged-matched controls	22.42 ± 1.55 *	10.11 ± 1.61 ng/mL *	
Aahmad et al. [76] 2018	30 preM women	24.58 ± 4.4 *	2667.3 ± 940.41 ng/L *	Preptin (whole-group): LS (r = 0.351, *p* = 0.041) and FN BMD (r = 0.312, *p* = 0.025), after adjustment for estradiol estradiol (r = 0.348, *p* = 0.006) age (r = −0.310, *p* = 0.016)
	30 postM women	24.95 ± 3.15 *	2102.27 ± 918.66 ng/L *	
Kaluzna et al. [77] 2021	36 HD + DM/IGT	27.1 (4.9) **	512 (1030.50) ng/L **	Preptin (whole group): LS BMD (r = −0.319, *p* = 0.01), FN Z-score (r = −0.241, *p* = 0.049) and total hip Z-score (r = −0.259, *p* = 0.034) HD vintage (r = 0.312, *p* = 0.007) PTH (r = 0.379, *p* < 0.001) osteocalcin (r = 0.262, *p* = 0.027) Preptin (DM/IGT group): BMD (r = −0.423, *p* = 0.014), FN Z-score (r = −0.499, *p* = 0.003) and total hip Z-score (r = −0.506, *p* = 0.002) HD vintage (r = 0.342, *p* = 0.041) PTH (r = 0.428, *p* = 0.009) osteocalcin (r = 0.347, *p* = 0.027) PTH > 200 pg/mL vs. PTH < 200 pg/mL: Preptin (ng/L) **: 695.5 (1184) vs. 452 (579) (*p* = 0.009) Osteocalcin (ng/mL) **: 230 (96) vs. 137 (178) (*p* = 0.001) ALP (U/L) **: 103 (64) vs. 75 (32) (*p* = 0.004)
	37 HD + NGT	23.3 (5.5) **	595 (788) ng/L **	
Preptin and bone and calcium metabolism				
El-Eshmawy et al. [57] 2015	50 overweight	27.5 ± 1.48 *	484.2 ± 50.84 pg/mL *	Preptin (overweight + obese group): Osteocalcin (β = −28.41, *p* = 0.04) after adjusting for BMI, fasting insulin, WC, HOMA-IR, cholesterol and triglycerides
	50 obese	33.3 ± 2 *	516.5 ± 66.98 pg/mL *	
	50 controls	23.9 ± 0.57 *	366.4 ± 38.53 pg/mL *	
Li et al. [78] 2018	102 non-CAC patients	25.83 ± 4.28 *	9.5 ± 3.91 ng/mL *	Preptin: Independent predictor of Agatston score (OR = 1.097, 95% CI: 1.021–1.179, *p* = 0.011) 2.9 times increased odds of an elevated CAC score in Q5 compared to Q 1–4 of serum preptin concentrations (OR = 2.913, 95% CI: 1.291–6.571, *p* = 0.01)
	118 CAC patients	25.42 ± 4.25 *	11.59 ± 7.81 ng/mL *	
Bebars et al. [79] 2019	30 rachitic children	NA	6.3 ± 1.5 ng/L *	Preptin (rachitic children): ALP (r = −0.97, *p* = 0.04)
	30 non-rachitic children	NA	8.3 ± 1.8 ng/L *	

ALP = alkaline phosphatase (B = bone, T = total), BMD = bone mineral density, BMI = body mass index, CAC = coronary artery calcification, DM = diabetes mellitus, FN = femoral neck, HD = hemodialysis, HOMA-IR = Homeostatic Model Assessment for Insulin Resistance), IGT = impaired glucose tolerance, LS = lumbar spine, NA = not available, NGT = normal glucose tolerance, PTH = parathormone, preM = premenopausal, postM = postmenopausal, WC = waist circumference. * mean ± standard deviation, ** median (interquartile range).

Preptin also differed according to menopausal status in the study of Aahmad et al. [76] (Table 2). Postmenopausal women had significantly lower BMD and both lower preptin and estradiol levels compared to premenopausal women, despite being matched by BMI. Preptin positively correlated with estradiol levels, but remained lower in postmenopausal women even after adjusting for estradiol levels. A significant positive correlation was found between preptin and lumbar spine and neck BMD, respectively, after adjustment for estradiol in both groups. Preptin was also negatively correlated with age in the whole group [76,79,80]. A recent article published in 2021 was the first to investigate the relationship between preptin and bone metabolism in hemodialysis patients [77] (Table 2). In a cohort of 73 hemodialysis (HD) patients with a mean HD vintage of 69.7 months, a median parathormone (PTH) of 227 pg/mL and median ALP of 92 U/L, preptin levels were similar in subjects with impaired glucose tolerance versus individuals with normal carbohydrate metabolism. However, serum preptin registered high inter-individual variability, with a mean of 1110.6 ng/L ± 1747 in the entire cohort. Preptin did not correlate neither with parameters of glucose metabolism, such as glucose, insulin or HOMA-IR, nor with BMI or body composition in HD patients. However, preptin was positively related to the HD vintage [77]. These rather discordant findings may be due to preptin accumulation in end-stage renal disease (ESRD) and over time.

Contrary to the previously published data, preptin negatively correlated with lumbar spine BMD, femoral neck and total hip Z-score both in the entire cohort and in the impaired glucose tolerance group [77]. This remains to be verified by other studies. The accumulation of preptin in ESRD may confound the association with BMD. The increased insulin secretion reported in ESRD as a compensatory mechanism for lower insulin sensitivity may also account for these unexpected findings [81]. Further on, a positive relationship was found between preptin and PTH and osteocalcin, respectively, in the whole group and impaired glucose tolerance group. When stratifying patients according to PTH values, patients displaying PTH > 200 pg/mL (and, thus, considered to have secondary hyperparathyroidism) had significantly higher preptin levels compared to patients considered to not have secondary hyperparathyroidism [77]. Thus, preptin may indirectly reflect bone turnover in HD patients that also associate impaired glucose tolerance, since no significant associations with bone mass or bone turnover were seen in HD patients with normal glucose tolerance. However, further studies are needed.

CKD-mineral and bone disorder (CKD-MBD) is a complex and severe complication of advanced CKD, associated with a high grade of morbidity and mortality. Bone disease in CKD is characterized by changes in bone turnover, mineralization and volume and is difficult to assess. The gold-standard for diagnostic is bone biopsy, which is rarely performed in clinical practice [82]. Therefore, the type of bone impairment is rather evaluated via bone turnover markers, PTH and bone densitometry [83,84]. The need for additional tools to optimize assessment of bone disease in CKD-MBD is high. Bone disease is strictly related to vascular calcifications in CKD-MBD [84,85].

### 4.3. Preptin: A New Player in Vascular Calcifications

Due to the reported associations between preptin and osteocalcin and knowing that osteocalcin favors vascular smooth muscle cells (VSMC) calcifications via Wnt signaling [86]—which is downstream signaling pathway for preptin [40]—one may assume that preptin may be involved in the pathogenesis of vascular calcifications. Indeed, in 2018, Li et al. [78] reported increased preptin levels in non-CKD patients with coronary artery calcifications (CAC) compared to non-CAC, despite their similar BMI (Table 2). Preptin independently contributed to the prediction of the Agatston score of CAC, together with age, male sex, hypertension, and β-blockers use. Also, the highest preptin concentration quintile had a significantly higher CAC score, being associated with 2.9-times increased odds of having an elevated CAC level compared to the other four quintiles [78]. The mechanism explaining high preptin levels associated with CAC is still to be clarified.

### 4.4. Preptin and Variants of Abnormal Bone Metabolism

Bebars et al. [79] found lower serum preptin levels in rachitic children compared to healthy controls. Preptin was inversely associated with ALP in rachitic patients, suggesting preptin may also mirror the severity of the disease, similar to ALP (Table 2) [79]. As rickets is characterized by increased bone turnover and low BMD [80], the evidence of accompanying low preptin levels agrees with previous studies reporting decreased preptin in conditions associated with low bone mass.

Khosla et al. [87] reported increased circulating levels of IGF2 E-peptide, the precursor of preptin, in hepatitis C-associated osteosclerosis (HCAO). HCAO is a rare clinical entity, with less than 30 cases described in the literature [88] since its first report in 1992 [89]. HCAO is characterized by increased cortical and trabecular bone, associated with increased bone density [88], which may be due to increased production of IGF2 and its binding protein IGFBP2 in the liver [87,90], or a possible imbalance in the RANK/RANKL/osteoprotegerin function, leading to osteoblast stimulation [91,92]. The clinical presentation comprises bone pain, especially in the lower limbs that can progress to the whole skeleton, weight loss and fractures. The increased bone formation rate is reflected by the high levels of serum ALP, leading to increased calcium accumulation in bone and a secondary increase in PTH secretion with reduced urinary calcium excretion [88]. Khosla et al. [87] demonstrated the isoform IGF2E_1–104_ derived from the full-length IGF2E_1–156_ is elevated in HCAO, a condition associated with high bone mass phenotype. Whether preptin also plays a role in the pathogenesis of HCAO is still unknown.

## 5. Preptin in Osteoporosis—Therapeutical Challenges

The anabolic bone effects of preptin became appealing to address with pharmacological treatment. However, Kowalczyk et al. [93] reported that an N-terminal shorter fragment of preptin—namely preptin_1–16_—retains the anabolic effects of preptin and is more attractive for the development of a preptin peptide analogue due to its smaller size. Unlike the full length molecule, the shorter 1–16 fragment has no activity over glucose metabolism [93]. However, due to its truncated form, it is enzymatically unstable [94]. Further on, Kowalczyk et al. [93] developed twenty-eight preptin_1–16_ analogues and assessed their effects upon the osteoblasts in vitro. They concluded that preptin_1–8_ is the shortest analogue that stimulated the formation of bone nodules and matrix mineralization in rat osteoblasts and would therefore be an appropriate target for chemical synthesis of new osteoporosis therapies [93].

From the same research group, two years later Amso et al. [94] reported the synthesis of second-generation analogues of preptin_1–16_ by incorporating hydrophobic non-proteinogenic amino acids at position 3 that had improved stability but lost the osteoblast proliferative effect. In a similar manner, the synthesis macrocyclic analogues resulted in the loss of the anabolic bone effects [94].

Whether preptin analogues could represent novel bone-anabolic agents for the treatment of conditions associated with low bone mass remains to be clarified. Another question to be answered in the future is the type of bone phenotype that could be targeted by preptin: every case of osteoporosis, or rather the low bone-turnover phenotype?

## 6. Future Directions: Bone Cancer

Assessing preptin in other conditions associated with disrupted Wnt/β-catenin and IGF signaling, such as osteosarcoma, may open new roads in the diagnostic approach of bone cancer.

Disruption of the Wnt signaling pathway has been described in the pathogenesis of osteosarcoma. However, controversies still exist, with both pro-oncogenic and tumor suppressor roles being reported [95]. Sclerostin is a soluble factor secreted by osteocytes that negatively regulates the Wnt/β-catenin signaling pathway [96]. Sclerostin silencing resulted in increased activation of the Wnt/β-catenin, leading to increased proliferation of osteosarcoma cells [97]. Recent data report anti-tumoral effects of sclerostin administration in animal models of osteosarcoma, where it inhibits the growth and migration of osteosarcoma cells [98]. On the other hand, sclerostin was found to be expressed [99] and upregulated in osteosarcoma [100]. Single-case genetic analysis from surgically removed sarcoma performed by Martson et al. [100] reported upregulation of sclerostin and Wnt inhibitory factor 1 (Wif1)—another Wnt/β-catenin antagonist—along with increased adiponectin expression in tumoral bone tissue. Moreover, mRNA expression of IGF1 and IGF2 in osteosarcoma cells were increased, while the insulin receptor (INSR) and insulin receptor substrate 1 (IRS1) genes were both downregulated [101]. Thus, Wnt bone activity is restructured during tumorigenesis and IGFs bone expression is also disrupted. As preptin upregulates Wnt/β-catenin signaling and is also linked to IGF2 activity, it merits further investigation as a marker for tumoral bone metabolism.

Moreso, aberrant alternative splicing of leptin receptor overlapping transcript (LEPROT) gene is also associated with osteosarcoma progression [102]. Leptin and preptin appear to regulate bone metabolism and osteocalcin expression in a dual manner, as described above. All these argue for preptin research in the pathogenesis of osteosarcoma.

## 7. Conclusions

Preptin is increased in conditions associated with insulin resistance and has anabolic bone effects in vitro and in vivo. It stimulates osteoblast differentiation, proliferation, survival and function. Peptin stimulates bone mineralization and positively regulates bone mass. The osteogenic effect of preptin is exerted via Wnt/β-catenin and ERK/MAPK signaling pathways in vitro. Preptin is reduced in ovariectomized rats, but increases after estradiol treatment, exercise training or alendronate administration.

In clinical studies, preptin is directly related to BMD in elderly men and pre- and postmenopausal women, although contradictory results were reported regarding the relationship between preptin and osteocalcin. The presence of insulin resistance may hinder the expected positive association, as osteocalcin is reduced in impaired glucose tolerance. Preptin is also reduced in rachitic children exhibiting low bone mass and also appears to be related to CAC. Finally, preptin may indirectly reflect bone turnover in CKD-MBD.

Whether preptin analogues could become an anabolic bone therapy still remains to be addressed.

## Figures and Tables

**Table 1:** Bone effects of preptin in experimental studies.

Research	Methods	Outcome
Xiao et al., 2019 [40]	Ob precursor cell line MC3T3-E1	Preptin upregulates Wnt/β-catenin pathway, RUNX2 and osteocalcin
Cornish et al., 2007 [47]	Primary rat Ob-like cell line	Peptin administration: Bone matrix deposition and mineralization in a dose-dependent manner Upregulates ERK/MAPK signaling pathway Antiapoptotic effects
	Adult male mice	Increased mineralization and bone surface after preptin administration
Liu et al., 2010 [52]	Human Ob	Proliferation of osteoblasts and increased ALP in a bell-shaped effect induced by preptin Preptin increases CTGF and activates ERK signaling pathway
Bosetti et al., 2013 [62]	Human Ob	Preptin: Stimulates proliferation to a lesser degree compared to IGF1 and IGF2 Did not increase ALP activity Induction of osteoclast differentiation and bone resorption activity, to a lesser extent compared to IGF1
Abdelfattah Abdulfadle K et al., 2023 [55]	Adult rats	Preptin: Decreased in OVX rats Increased in OVX + Estradiol, OVX + Ex, OVX + Alendronate, OVX + Estradiol + Ex

ALP = alkaline phosphatase, CTGF = connective tissue growth factor, Ex = exercise IGF = insulin-like growth factor, Ob = osteoblasts, OVX = ovariectomized.
